# Encouraging classroom activities for children using avatar robots: a field trial

**DOI:** 10.3389/frobt.2025.1571804

**Published:** 2025-09-09

**Authors:** Megumi Kawata, Masashi Maeda, Hirokazu Kumazaki, Hiroko Kamide, Jun Baba, Naomi Matsuura, Hiroshi Ishiguro, Yuichiro Yoshikawa

**Affiliations:** 1 Department of Engineering Science, Graduate School of Engineering Science, Osaka University, Osaka, Japan; 2 Yonenosho Elementary School, Matsusaka, Mie, Japan; 3 Department of Neuropsychiatry, Nagasaki University Graduate School of Biomedical Sciences, Nagasaki, Japan; 4 Graduate School of Law, Kyoto University, Kyoto, Japan; 5 AI Lab, Department of Artificial Intelligence, CyberAgent, Inc., Tokyo, Japan; 6 Department of Education, Elementary School Attached to Mie University Faculty of Education, Tsu, Mie, Japan

**Keywords:** encouraging classroom activities, remote-controlled avatar, multiple operators, field trial, educational support

## Abstract

Educational institutions are facing a critical shortage of teachers worldwide. Consequently, the trend of introducing interactive robots into educational sites is growing. However, most previous research focused on specific subjects or time slots, and only a few studies have introduced interactive robots to participate in whole classroom activities with children routinely. This study investigates the use of avatar robots operated by multiple remote operators in elementary school classrooms. Over nine days, a 5th-grade class was observed to assess the robot’s impact on student engagement, motivation, and peer interactions, and compared to classes where any avatar robots were not introduced. Key findings include improved student confidence in presentations, enhanced social interactions during recess, and positive feedback on the robot’s role in supporting classroom activities. The results suggest that avatar robots, with consistent remote operation, can provide valuable educational support without strong negative reactions from students.

## 1 Introduction

In recent years, the shortage of teachers has become a severe problem in the Japanese educational field (Ministry of Education and Culture and Sports and Science and Technology, 2022). Teacher shortages are a global issue, as highlighted in the report by OECD (Organisation for Economic Co-operation and Development, 2020). Excessive workload on teachers and US and UK government policies (Perryman and Calvert, 2020; Ingersoll et al., 2016) has been shown to lead teacher resignations (Ingersoll and Smith, 2003). Furthermore, declining social status of teachers and low salaries (Wiggan et al., 2021; Han et al., 2018) are considered to cause the reluctance of the young generation to the teacher profession.

In response to these challenges, researchers and policymakers are increasingly recommending the utilization of community resources to support education (Giraudeau and Bailly, 2019; Canedo-Garcia et al., 2017). Initiatives, where older adults support young people in educational settings, are being introduced worldwide (Newman and Hatton-Yeo, 2008). Such an initiative is advantageous because it also ensures opportunities for social activities for older adults (Galbraith et al., 2015). Collaboration between schools and communities have been promoted by the governments in Japan (Ministry of Education and Culture and Sports and Science and Technology, Report of the Central Council for Education, 2015) as well as in other countries (Newman and Hatton-Yeo, 2008). However, several practical problems have also been highlighted, including the time constraints of educational supporters, restrictions on the range of movement of elderly educators owing to the aging-related decline in physical abilities, and confusion experienced by children with outsiders (Newman and Hatton-Yeo, 2008; Murayama et al., 2019), which makes it difficult to supply necessary human resources. Governmental support for school-community collaboration such as providing sufficient budget and human resources has been concerned to be insufficient in Japan (Shimojo, 2020). Meanwhile, there is another concern for children to experience confusion with outsiders in such a collaboration (Newman and Hatton-Yeo, 2008; Murayama et al., 2019). In Japan, lack of consensus to treat outsiders (i.e., community supporters) in school has been pointed out to enhance this confusion (Yoshida and Yoshizawa, 2024). On the other hand, the telepresence robot is expected to allow users to work against these constraints, and therefore has been attempted to allow people to engage in educational support from a distance (Kasuk and Virkus, 2024).

In recent years, increasing attention has been focused on using avatars to enable individuals to work from different locations and provide services remotely, thereby overcoming spatial and time constraints (Ishiguro, 2021; Baba et al., 2020; Nakanishi et al., 2021). The use of social robots as avatars is gaining increasing interest in educational contexts because of the expectation that children may feel less resistant when interacting with them. This has led to an increase in the number of initiatives that incorporate avatars into educational settings (Belpaeme et al., 2013; Nakanishi et al., 2022). For example, Komatsubara et al. developed a science learning support system using a semi-autonomous robot with a remote operator to provide science-related dialogues during recess (Komatsubara et al., 2015). Yun et al. demonstrated that a robot named EngKey, which was introduced in 29 elementary schools for 3 months and was remotely operated by a teacher in the Philippines to teach English to South Korean students, could improve the grades of students (Yun et al., 2011). Moreover, Hashimoto et al. showed that children were highly motivated to participate in class when an android robot called SAYA, which was operated by a remote operator who acted as a teacher, was used in a classroom (Hashimoto et al., 2011). In previous studies, avatar robots have been used for limited tasks, for example, as classroom teachers in specific classes (Hashimoto et al., 2011; Tanaka et al., 2013) and as teaching assistants in specific classes (Yamazaki et al., 2012; Nakanishi et al., 2022). Most of the research has been focused on class time, which is not recess. By contrast, the systematic review by Hodges et al. confirmed numerous benefits of recess for children, including academic and cognitive benefits, behavioral and emotional benefits, physical benefits, and social benefits, which can collectively improve their academic performance (Hodges et al., 2022). Although related research has introduced robots during recess (Kanda et al., 2007), previous studies neither supported entire classroom activities, including during recess nor did they support interpersonal relationships, which cannot be established (Murayama et al., 2019), through the sequence of whole classroom activity. Only one operator was involved in these studies; however, this approach may not be practical when supporting classroom activities for an extended period. By contrast, in a previous study, we introduced avatar robots operated by multiple remote operators in rotation to assist whole classroom activities (Kawata et al., 2023). More specifically, an interactive robot participated in whole classroom activities for 3 days and interacted with children freely. Children did not show negative reactions to the avatar robot operated remotely by multiple individual operators rotationally. Nonetheless, the effects of introducing avatar robots into classrooms remain unclear. Intermittent implementation may not provide sufficient interaction between the avatar robot and the children to assess these consequences.

In this study, we expanded the settings of previous research (Kawata et al., 2023) to examine the effects of a long-term introduction of an avatar robot into an elementary school classroom. Specifically, when planning for the long-term deployment of an avatar robot, frequent changes in remote operators are expected. We implemented an information-sharing strategy to maintain consistent behavior and character across operators. We believe that this strategy could help reduce any communication inconsistencies that may arise with the children owing to shifts in remote operators. Additionally, we implemented group-focused communication strategies to ensure that each child in the class had equal interaction opportunities with the avatar robot. The avatar robot was continuously introduced to a class for fifth graders of the Mie University-affiliated elementary school for 9 days from 4 to 14 July 2022, excluding public holidays, to rotate classroom groups. In Japan, fifth graders are 10–11 years old. This class was chosen as a normal one in the public school. Three operators alternately operated the avatar robot. We assessed the impact on the children and the classroom before and after its introduction. The results suggest that daily and long-term interactions with an avatar robot not only provide children with comfortable recess and classes but also potentially encourage their participation in classroom activities. Based on these findings, we examined the character and behavior of the robot, including the appropriateness of the remote operator system. We discuss the potential for educational support using the avatar robot.

## 2 Related works

### 2.1 Educational support using telepresence robots

Telepresence robots are robotic devices that can be operated from a remote location. These robots incorporate video conferencing equipment (Kristoffersson et al., 2013). Specifically, telepresence robots have cameras, speakers, microphones, screens, and sensor-based motion control, enabling operators to log in and remotely control the robot from a tablet or PC. Operators can use the cameras on a device to access and monitor the local environment. Telepresence robots can be used to provide social interaction from a distance (Kristoffersson et al., 2013). Telepresence robots are being increasingly deployed in real-world educational settings to help children learn (Conti et al., 2017; Woo et al., 2021; Botev and Rodríguez Lera, 2021; Shimaya et al., 2019).

Telepresence robots in education have shown the potential to support children’s learning. Reportedly, students’ grades improved when a native English-speaking human teacher operated a robot to teach English to children from a remote location (Yun et al., 2011; Park et al., 2011). A teleoperated robot operated by an English teacher was introduced to language education and compared with a Skype interface (Tanaka et al., 2013). This suggested that the robot facilitates communication between the teacher and participants with potentially beneficial educational effects. By contrast, it has been shown that even if the remote operator is not an education specialist, the robot could make children interested in the class. Hashimoto et al. installed the Android SAYA in a classroom as a teacher, and one operator remotely operated the robot to conduct during a class. The approach piqued the children’s interest in science classes (Hashimoto et al., 2011). A teleoperated robot operated by Australian children was introduced to language education, and it was observed that Japanese students actively used English phrases to talk to the robot (Tanaka et al., 2013). However, these studies have primarily focused on providing learning support in specific subject areas, with less emphasis on activities that require long-term, continuous intervention, such as fostering interpersonal relationships within the classroom.

### 2.2 Supporting classroom activities

Teachers should make students participate in the learning process to optimize each student’s learning and development and to prevent gradual withdrawal, truancy, and dropout (Havik and Westergård, 2020). Additionally, positive interpersonal relationships between teachers and students positively impact learning, motivation, etc. (Xie and Derakhshan, 2021). In particular, positive attention (Frisby, 2019) and praise for student language use (Chalk and Bizo, 2004; Havik and Westergård, 2020) have been reported to be effective in promoting classroom participation behaviors. However, owing to teacher shortages, students may lack the support to actively participate in classroom activities (García and Weiss, 2019; Sutcher et al., 2019). Consequently, attempts have been made to support this need through technology. Among these, initiatives that promote the autonomy of children have been reported. For example, Nakanishi et al. introduced a small humanoid robot called Sota, which an older adult remotely controlled. It was shown that children began to talk about themselves spontaneously when the robot interacted with them in the morning and on their way home (Nakanishi et al., 2022). In another experiment, a teleoperated robot called Telenoid in an elementary school classroom for 2 days promoted communication among children, who showed more active attitudes toward group work (Yamazaki et al., 2012). Thus, the interaction of children with a robotic mediator during group work resulted in more active children. However, these studies were limited to support related to specific classroom activities, they did not address daily whole-class activities or full-day support for classroom activities comprising multiple participants and times. Studies demonstrated that providing children with comfortable breaks is crucial for aspects such as developing relationships and facilitating learning (Ramstetter et al., 2010; Bauml et al., 2020; Stapp and Karr, 2018). Furthermore, no study evaluated whether such support positively impacts classroom activities and children’s ability to socialize and communicate. Therefore, although technological interventions such as teleoperated robots are expected to enhance student engagement and participation in certain activities, it remains unclear how these technologies will support class and recess time on a daily basis.

## 3 Interactive avatar robot

### 3.1 Avatar robot system

**FIGURE 1:**
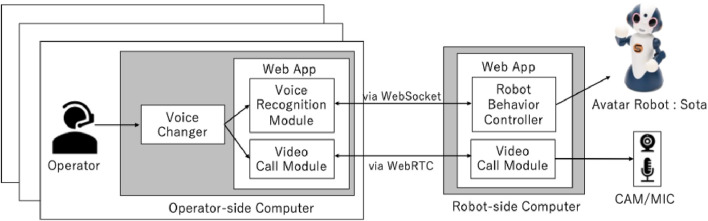
Structure of an interactive teleoperation robotic system, based on a study by Baba et al. (2020). Using the voice changer, the audio output from the avatar robot remains consistent even when multiple remote operators are switched.

Based on a previous study by Baba et al. (2020), multiple operators remotely controlled a social interactive humanoid robot called Sota using a teleoperation system called “Sota100” to achieve flexible communication with children and teachers. Sota100 has proven its performance and feasibility in various fields (Baba et al., 2020; Hatano et al., 2023; Song et al., 2021; Okafuji et al., 2024), including environments with children (Nakanishi et al., 2022), which indicates its design to be well accepted even in the classroom situation. The system configuration of the avatar robot used in this study is shown in Figure 1. The avatar robot Sota was developed by Vstone Corporation. Sota is a tabletop communication robot (280 mm (H) × 140 mm (W) × 160 mm (D)). It has eight degrees of freedom (one axis for the body, two axes for the arms, and three axes for the neck). The operators remotely controlled the robot using a web application with video-calling capabilities called Web Real-Time Communication. They viewed the video stream obtained using a wide-angle web camera located at the back of the robot, listened to audio recorded in the classroom using an external microphone (YAMAHA, YVC-1000), and engaged in conversations with the children in the classroom using a speakerphone mounted at the front of the robot base. A mini-PC installed inside the base could execute a video-calling web application program and enable the transmission of control commands to the robot. The system interface was tested by older adult operators in a study by Nakanishi et al. (2022) and it proved easy to use, even for older adults. The mini-PC was connected to the network of a Mie University-affiliated elementary school using a wired local area network cable.

### 3.2 Interactive behaviors

Children had classes from the first period to the fifth period everyday. The avatar robots participated in classroom activities from the first recess after the first class to the long recess after the fourth class (9:30 a.m. to 1:25 p.m.); an operator was assigned to the first slot from the first recess (9:20 a.m.) to the end of the third class (11:30 a.m.). Subsequently, another was assigned to the second slot from the recess after the third class (11:30 a.m.) to the end of long recess after the fourth class (13:25 p.m.). Note that the robot avatar was tentatively suspended during the lunch and cleaning up time in the long recess. Each operator should log in to the control system 10 min before the start of their assigned slot, review the interaction history logs, and prepare for the upcoming interaction. The avatar robot was assigned the character “I am attending this class because I want to go to elementary school again” to give the children the impression that the avatar robot is motivated to participate in classroom activities. We did not disclose to the children that a human operator was remotely controlling the avatar robot. During classes, it spoke in response to requests from the children or the teacher. For example, the teacher sometimes requested it to respond by saying “what do you think about this opinion (of the children)?” and it praised or empathized by saying “I see, that’s one way of thinking!”, “I think so too!” or “I wish you would tell me more about it!” In the group work, the operators would call children’s names to solicit their opinions and encourage them to exchange opinions. Additionally, comments by the children were met with words of praise and nods of agreement (e.g., “What do you think, [name]?”, “You’ve expressed your opinion brilliantly!” or “Thank you for explaining that so clearly!”). During recess, basically, it did not only answer questions from the children but also freely asked the children various questions related to their school life, friendships, and understanding of the lessons. It sometimes offered to review any challenging parts when necessary. Furthermore, the operator occasionally asked other children to help involve those who had not spoken much with the avatar robot. When communicating with the children, the operator attempted to listen attentively without attacking or criticizing them, while protecting their privacy and avoiding comments that might impose gender roles or harassment. Note that its gaze direction was manually controlled by the operator in real time so as to look like it was directed to a child or the teacher. Its gestures were triggered to enhance its reactions, such as raising both hands and nodding.

### 3.3 Information sharing

Multiple operators were operating the robot; we needed to share information to avoid inconsistencies in communication. Inconsistencies might occur owing to differences in how each operator interacts with the children or owing to forgetting previous interactions. If the robot cannot maintain a consistent character, this could lead to distrust and confusion among children and hinder the goal achievement. Therefore, we used Google Forms and Google Spreadsheets. The operators recorded observations of the children’s actions and their responses during class. Ten minutes after the start of each class, 5 minutes were allocated for recording, inputting the collected information into Google Forms, and submitting it. These records were then submitted and reviewed by the next operator on a shared Google Spreadsheet before their session began, ensuring consistent communication and responses. This ensured consistency in information sharing and communication reactions among remote operators. This tool can share a log of interaction by all operators.

Additionally, real-time information sharing with the classroom teacher was facilitated through Slack. Slack, a group communication tool, supported interactions across devices, such as PCs and smartphones, enabling voice calls and file sharing. The classroom teacher uploaded images of the blackboard and textbook content to Slack, which remote operators used as references to participate in the lessons alongside the children.

## 4 Methods

### 4.1 Overview of the method

In this experiment, the interactive avatar robot “Sota” was installed in a fifth-grade elementary school classroom. The avatar robot, with three remote operators, participated in the class for 9 days, from 4 to 14 July 2022, excluding holidays. The remote operators interacted with the children and teachers via the robot. In addition, its position in the classroom was changed such that all groups comprising four children had equal chances of sitting next to it. During class, the avatar robot called out the names of the children to motivate them to share their opinions, responded to their ideas, and praised them. During recess time, the avatar robot focused on the members of the target group, which was situated beside it. The robot asked them about their understanding of the lesson, suggested that they review it, and encouraged conversation among the children. During lunch breaks, the robot engaged in free conversations with children from different groups, as they spontaneously gathered around the robot. These interactions explored whether the avatar robot provided comfort during recess time, encouraged the children to share their opinions and presentations during class, and supported their classroom activities. Moreover, we conducted a questionnaire survey to compare children’s sentiments regarding the avatar robot; the activities performed during recess time and in the classroom were also compared between the classes with and without the avatar robot, and the relationship between these factors was also examined. Furthermore, we used the Hyper-QU questionnaire (Musashi and Kawamura, 2015) to analyze changes in the classroom atmosphere and social skills of the children when the avatar robot was installed.

### 4.2 Participants

The participants of this study included 96 children in the fifth grade of an elementary school attached to a Mie University-affiliated elementary school. There were three fifth-grade classes, and Sota was installed in only one classroom, that is, class 5C (N = 32), which was considered the experimental group. Conversely, the other two classes without an avatar robot (classes 5A and 5B (N = 64)) were considered as the control group. Note that the children of class 5C participated in preliminary experiments before the main experiment (Kawata et al., 2023).

### 4.3 Apparatus

The avatar system described in 3.1 was utilized. The location of the avatar robot was changed daily to provide all the children with an opportunity to interact with the avatar robot. The remote operators used a laptop (Lenovo IdeaPad Core-i7 SSD 512GB) and a headset (Jabra 75 Evolve). Moreover, Morphvox (ScreamingBeeInc, 2024), a voice-changing software, was used to apply a ‘child’ effect to the voice of the operators, resulting in similar voice sounds for all the operators and ensuring a seamless transition between them.

### 4.4 Measurements

We used Kamide’s index to evaluate children’s sense of comfort and stress when spending time with the avatar robot, as well as their ability to interact with it—corresponding to (1) sense of wellbeing in the presence of the robot (Kamide et al., 2015). Subsequently, Kamide’s index was employed to assess the avatar robot’s personality and whether it exhibited consistent responses, aligning with (2) independent behavior of the robot (Kamide et al., 2016). Additionally, we created a questionnaire to determine whether the children felt comfortable during recess, which relates to (3) evaluation of comfort during recess time. Finally, we developed a questionnaire asking whether the children were comfortable giving presentations about the class time they spent with the avatar robot, corresponding to (4) evaluation of ease of presentation. The questionnaire is detailed in Table 1. Moreover, we investigated the effects of the avatar robot only for children in the class where the avatar robot was installed using the Hyper-QU questionnaire, which was used as a standardized classroom group assessment (Musashi and Kawamura, 2015).

**TABLE 1:** Items in the questionnaire presented to the children.

Comfort	1. I Feel peace of mind when with the robot
	2. I feel relieved
Peace of mind	3. When I am with this robot, I feel restless
	4. This robot makes me feel stressed out
Performance	5. This robot seems to respond to my questions
	6. I think this robot recognizes its surroundings
Controllability	7. This robot is unlikely to harm human body
	8. The robot is unlikely to become uncontrollable
Self-consistency	9. I think this robot has self-consistency
Consistent responses	10. I think this robot gives consistent responses
Human Nature Positive	11. I think this robot is a fun-loving robot
	12. I think it is a sociable robot
Human Nature Negative	13. I think this robot is impatient
	14. I think this robot is aggressive
Uniquely Human Positive	15. I think this robot is polite
	16. I think this robot is mad
Uniquely Human Negative	17. I think this robot has poor thoughts
	18. I think this robot is rude
Comfort During Recess	1. I feel comfortable during recess
	2. It is easy to talk to my classmates during recess
Presenting in class	1. It is easy to present a problem and obtain a clear answer
	2. It is easy to present a problem and obtain an unclear answer
	3. It is easy to present to a small group
	4. It is easy to present in front of the entire class

#### 4.4.1 Sense of wellbeing in the presence of the robot

This dimension assessed the sense of wellbeing experienced by the children in the presence of the robot in the classroom. The questionnaire was developed based on the sense of security scale used by Kamide et al. (2015). We chose the two items with the highest factor loadings in the different categories, including evaluations of “Comfort,” “Peace of Mind, ” “Performance,” and “Controllability.” Responses were collected on a seven-point scale ranging from 1 (“Strongly Disagree”) to 7 (“Strongly Agree”).

#### 4.4.2 Independent of the robot

This dimension consisting of totally 10 items evaluated the perceptions regarding the character of the avatar robot. In this experiment, there is a possibility that children might perceive inconsistencies in the avatar robot’s behavior due to the fact that it was operated by multiple individuals with different personalities. Such inconsistency could lead to the impression that the robot did not have consistent awareness about itself. Therefore, we decided to ask the children directly about their perceptions of the robot’s self-consistency by using the following two question items: “This robot seems to have self-consistency” and “I infer the robot’s responses are consistent.” Similar to the previous dimension, a seven-point scale was used for responses. Additionally, potential self-inconsistency was considered to also reduce the impression of the robot’s anthropomorphism. Based on the previous research developing the Japanese version of psychological scales to evaluate anthropomorphism (Kamide et al., 2016), four sub factors for it, namely “Human Nature Positive,” “Human Nature Negative,” “Uniquely Human Positive,” and “Uniquely Human Negative.” Two question items with higher factor loding were chosen to assess each sub factor.

#### 4.4.3 Comfort during recess

This category assessed whether children experienced comfort during recess and whether they experienced ease while engaging with their classmates. It comprised two questionnaire items: “I feel comfortable during recess” and “I find it easy to talk to my classmates during recess.” Responses were collected on a seven-point scale ranging from 1 (“Strongly Disagree”) to 7 (“Strongly Agree”).

#### 4.4.4 Presenting in class

This dimension evaluated the ease of making presentations in four different scenarios during class. The questionnaire contains four items: “It is easy to make presentations for problems with clear answers,” “It is easy to make presentations for problems with unclear answers,” “It is easy to make presentations in small groups,” and “It is easy to present in front of the entire class.” Responses were collected using a seven-point scale ranging from 1 (“Strongly Disagree”) to 7 (“Strongly Agree”).

#### 4.4.5 Total assessment in classroom

The impact of the avatar robot on the classroom was evaluated by adopting items from the Hyper-QU questionnaire developed by Musashi and Kawamura (2015), which measures the satisfaction of each child with school life, motivation for class activities, and social skills of the children. In particular, the questionnaire measured the extent to which children felt that their classmates and teachers approved of their presence and behavior (approval or disapproval), and evaluated their satisfaction with school life. Moreover, the questionnaire assessed the motivation of the children for class activities by measuring their motivation and satisfaction (friendship, motivation to learn, and class atmosphere). Furthermore, the social skills required for interpersonal relationships necessary for group formation were measured (consideration and interaction). Consideration indicates whether basic manners and rules were observed in adult interactions, while interaction indicates whether children were actively involved with their friends. We assessed the extent to which children mastered these social skills. These categories were used to provide a comprehensive study of the impact of avatars in the classroom environment.

### 4.5 Procedure

**FIGURE 2:**
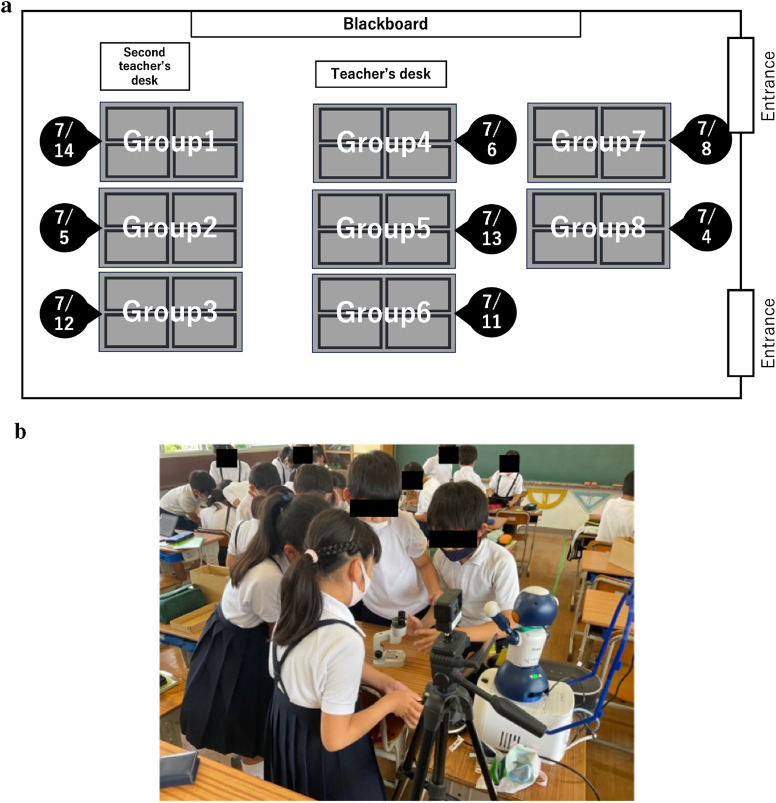
**(a)** Schedule of the movement of the avatar for 2 weeks. **(b)** Interaction between the avatar and children of Group 5 during class. **(a)** This figure shows the schedule and movement of the avatar over 2 weeks. The classroom is divided into eight groups, with the numbers in black balloons indicating the dates the avatar was positioned in each group. **(b)** This image shows children interacting with the avatar robot. This image was taken during a science class and shows children of Group 5, explaining the microscope to the avatar robot.

The avatar robot “Sota” was placed on a specialized desk for children and moved to a different group each day for 9 days. The classroom arrangement for 5C is shown in Figure 2a. As shown in Figure 2b, Sota interacted closely with the children in their assigned groups during the class. Note that during recess and lunch breaks, children outside of the target group could also interact freely with the robot every day. On the fourth day, when class participation was low because of the use of other facilities in the school, Sota was placed on the desk of the teacher. Two remote operators were assigned each day to work in shifts during the first and second halves of the designated period. The remote operators comprised two staff members from Osaka University and one staff member from Nagasaki University. They used a remote-control interface from their respective laboratories to operate Sota. The avatar robot was operated only when the children used their home classroom for classes. The total number of times when it was operated in the class was 16 over nine days. To avoid disrupting the flow of the class, operators were instructed not to have the robot speak during the class and to refrain from initiating any utterances until called upon. On the other hand, it freely interacted with the children during recess. Three operators were randomly assigned to the slots for operating the avatar robot for 8, 5, and 3 periods, respectively. Slack was used as a tool to facilitate real-time communication between the classroom teacher and remote operators. The classroom teacher shared photos of the blackboard content and textbook material using Slack. The remote operators referred to information from the textbooks and handouts shared via Slack and participated in the class. The designated remote operator documented the communication that occurred between the teacher, children, and themselves using Google Forms and shared this information with the other remote operators. Questionnaires assessing the appropriateness of the avatar robot and examining the potential of the avatar robot to support education for both the experimental and control groups were administered on July 1st as a pretest and July 15th as a posttest. Moreover, the “HyperQU” survey to evaluate the impact on the classroom was administered only to the experimental group on 1st July as a pretest and on 15th July as a posttest. Additionally, on 15th July, the experimental group provided free descriptive answers regarding their impression of their two-week-long interactions with the robot. Following the questionnaire, we observed the discussion on “how to interact with the robot” in the moral class for the children of the experimental group.

## 5 Results

We used a mixed-design two-factor analysis of variance (ANOVA) with a variable of the period (pretest/posttest) as the within-subject factor and a variable of the robot (with/without) as the between-subject factor when analyzing the measurements in the four categories. We analyzed the questionnaire responses of 30 children in the experimental group and 44 children in the control group who answered both pretest and posttest for the different categories, such as their sense of wellbeing in the presence of the robot, independence of the robot, comfort during recess time, and ease of presentation. Table 2 presents the test results for the main effects and interactions for each question. We conducted paired t-tests to analyze the differences between the two time points, that is, 1st July (pretest) and 15th July (posttest), concerning the classroom with the avatar. The analysis aimed to assess the school life motivation scale for the subscales of friendship, learning motivation, classroom atmosphere, school life satisfaction related to approval/disapproval of their classmates, and social skills based on consideration and interaction. We analyzed 29 children from a total of 34 children in the experimental group who responded to both the pretest and posttest assessments.

**TABLE 2:** Mean (standard deviation) of scales related to the following questionnaire items: sense of wellbeing in the presence of the robot, independence of the robot, comfort during recess, ease of presenting.

Evaluation category		Experimental group		Control group		Main effect		
		Pretest	Posttest	Pretest	Posttest	Robot	Period	Interaction
Sense of Wellbeing in the Presence of the Robot	Comfort	6.7 (0.5)	6.9 (0.4)	5.1 (1.6)	5.4 (1.7)	0.00*	0.08	0.53
	Peace of mind	1.5 (0.9)	1.6 (1.3)	2.1 (1.3)	1.9 (1.1)	0.06	0.92	0.39
	Performance	6.4 (1.1)	6.6 (0.8)	5.4 (1.3)	5.5 (1.4)	0.00*	0.24	0.74
	Controllability	6 (1.9)	6 (1.8)	5.8 (1.5)	5.7 (1.8)	0.53	0.93	0.89
Independence of the Robot	Self-consistency	5.6 (1.87)	5.9 (1.8)	5.2 (1.7)	5.3 (2.0)	0.19	0.26	0.78
	Consistent responses	5.7 (1.53)	5.9 (1.6)	5.0 (1.4)	4.8 (1.8)	0.006*	0.93	0.42
	Human Nature Positive	6.3 (0.9)	6.6 (0.7)	5.4 (1.2)	5.7 (1.4)	0.00*	0.01	0.94
	Human Nature Negative	1.6 (1.1)	1.4 (0.8)	2.1 (1.2)	2 (1.2)	0.02	0.26	0.79
	Uniquely Human Positive	6 (1.0)	6.2 (1.0)	5.2 (1.2)	5.2 (1.0)	0.00*	0.21	0.49
	Uniquely Human Negative	1.5 (1.0)	1.3 (0.5)	2.5 (1.4)	1.9 (1.1)	0.00*	0.00*	0.24
Comfort During Recess	Recess	6.1 (1.1)	6.6 (0.8)	6.2 (1.0)	6.2 (1.4)	0.39	0.11	0.098 †
Presenting in class	Clear answers	5.8 (1.7)	6.2 (1.3)	5.9 (1.9)	5.6 (2.1)	0.56	0.76	0.01*
	Unclear answers	4.7 (2.0)	4.7 (2.1)	4.2 (2.1)	4.1 (2.2)	0.24	0.92	0.77
	In a small group	6 (1.2)	6.5 (0.9)	5.9 (1.6)	5.9 (1.8)	0.33	0.09	0.04*
	Front or the entire class	5.3 (1.7)	5.4 (1.7)	4.4 (2.3)	4.5 (2.2)	0.05*	0.66	0.93

An asterisk indicates significance at the Bonferroni-adjusted significance level, and a dagger ( † ) indicates a significance trend at the Bonferroni-adjusted significance level.

### 5.1 Sense of wellbeing in the presence of the robot

For each measurement, the mixed-design ANOVA was conducted with the adjusted significance level as per the Bonferroni method ( α = 0.013 = 0.05/4) considering the number of measurements for the children’s sense of wellbeing in the presence of the robot. Regarding the results for comfort, a significant main effect of the robot factor (F (1,72) = 30.073, p< 0.01, $\eta {\text{p}}^{2}$ = 0.295) was exhibited. Regarding the results for performance, a significant main effect of the robot factor (F (1,72) = 16.274, p< 0.01, $\eta {\text{p}}^{2}$ = 0.184) was exhibited.

### 5.2 Independence of the robot

For each measurement, the mixed-design ANOVA was conducted with the adjusted significance level according to the Bonferroni method ( α = 0.008 = 0.05/6), considering the number of measurements for independence of the robot. For the results related to consistent responses, a significant main effect of the robot factor (F (1,72) = 7.94, p = 0.006, $\eta {\text{p}}^{2}$ = 0.1) was found. Regarding the results related to the dimension of human nature positive, a significant main effect of the robot factor (F (1,72) = 13.22, p = 0, $\eta {\text{p}}^{2}$ = 0.09) was exhibited. For the uniquely human positive results, a significant main effect of the robot factor (F (1,72) = 17.434, p = 0, $\eta {\text{p}}^{2}$ = 0.195) was exhibited. For the uniquely human negative results, significant main effects of the robot factor (F (1,72) = 12.942, p = 0, $\eta {\text{p}}^{2}$ = 0.152) and survey period factor (F (1,72) = 8.987, p = 0.003, $\eta {\text{p}}^{2}$ = 0.111) were exhibited.

### 5.3 Comfort during recess

We combined the results of the two questions related to the recess and analyzed them using a mixed-design ANOVA. The robot (F (1,72) = 0.761, p = 0.385, $\eta {\text{p}}^{2}$ = 0.011) and period factors (F (1,72) = 2.555, p = 0.114, $\eta {\text{p}}^{2}$ = 0.034) were not statistically significant. However, a trend towards significance in the interaction (F (1,72) = 2.807, p = 0.098, $\eta {\text{p}}^{2}$ = 0.038) was observed. The simple main effects of the robot factor were not significant with the period factor. However, the simple main effect of the period factor was significant with the with-robot condition (F (1,29) = 5.85, p = 0.02, $\eta {\text{p}}^{2}$ = 0.057). Specifically, in the class with the robot (5C), the pre-test scores (M = 6.1, SD = 1.1) were lower than the post-test scores (M = 6.6, SD = 0.8).

### 5.4 Presenting in class

The category of ease of presentation consisted of four different questions related to different situations. For questions related to clear answers for ease of presentation, a significant interaction was observed (F (1,72) = 7.142, p = 0.009, $\eta {\text{p}}^{2}$ = 0.09). The simple main effects of the robot were not significant with the period factor. However, the simple main effect of the period factor was significant in the class with the robot (F (1,29) = 6.0, p = 0.02, $\eta {\text{p}}^{2}$ = 0.019). Specifically, in the class with the robot (5C), the pretest scores (M = 5.8, SD = 1.7) were lower than the posttest scores (M = 6.2, SD = 1.3). Regarding the results of presentations in small groups, a significant trend for the period factor (F (1,72) = 2.903, p = 0.092, $\eta {\text{p}}^{2}$ = 0.039) was observed. Moreover, a significant interaction was observed between them (F (1,72) = 4.18, p = 0.044, $\eta {\text{p}}^{2}$ = 0.055). The simple main effects of the robot factor were not significant at any level of the period factor. However, the simple main effects of the period factor were significant in the class with the robot (F (1,29) = 6.0, p = 0.021, $\eta {\text{p}}^{2}$ = 0.02). Specifically, in the class with the robot (5C), the pretest scores (M = 6.0, SD = 1.2) were lower than the posttest scores (M = 6.5, SD = 0.9). For presentations in front of the entire class, a significant main effect of the robot factor (F (1,72) = 4.125, p = 0.046, $\eta {\text{p}}^{2}$ = 0.054) was observed.

### 5.5 Total assessment in classroom

**FIGURE 3:**
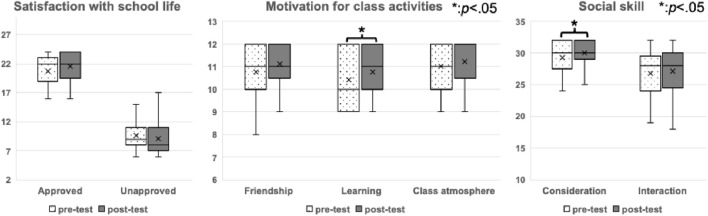
Results for the categories of satisfaction with school life, motivation for class life, and social skill according to Hyper-QU. The results are summarized in Panel 3 for the 29 children of the experimental group, who responded to both the pretest and posttest assessments.

Regarding learning motivation, a significant difference was observed between the means of both levels (t = 2.167, p = 0.038), with a pretest mean (M = 10.4, SD = 1.2), which significantly smaller than the posttest mean (M = 10.8, SD = 1.0). For the aspect of consideration, a significant difference was observed between the means of both levels (t = 2.178, p = 0.038), with a pretest mean (M = 29.3, SD = 2.4), which is significantly smaller than the posttest mean (M = 30.0, SD = 2.0), suggesting improved adherence to basic manners and rules in interactions over time (Figure 3).

## 6 Discussion

### 6.1 Characterization and behavior of the avatar

First, the results of this research show that after 9 days of interaction, the children in the classroom with the avatar robot felt it easier to present questions for which children knew that the answers were clear. Moreover, they indicated that the children felt more comfortable presenting in front of small groups. Additionally, learning motivation was increased. The strategies adopted for the avatar’s behavior, which included calling out children by name, encouraging the expression of personal opinions, and praising presenters, are likely to have encouraged children to learn through active class participation. In other words, the implemented avatar robot was able to be operated by multiple operators and could effectively support children’s classroom activities, as intended to be checked in this experiment. We found consistent positive comments on the perceived impact of the avatar robot on their classes in free descriptive feedback from the children, such as, “Having the robot in my group made it easier for me to participate more in the class,” “Having the robot made it easier to present during the class,” and “Having the robot in class boosts the classmates’ motivation to learn, making it more enjoyable.” They are consistent with related studies showing that introducing an avatar robot into a community of children made children more active (Yamazaki et al., 2012).

Second, the results show that interactions with the avatar robot enabled children to spend their recess time more comfortably, and it seemed to promote easier communication with their classmates. During recess time, the adopted strategy for interaction involved free conversations with children on various topics, such as classroom atmosphere, friendship, and school life. Such interactions are considered to provide comfort to children during recess time. The manner in which the avatar mediated the interactions between children was also observed during recess time, suggesting the potential role of the avatar in promoting interpersonal interactions. The children provided consistent answers in the free description of their impression of their time with the avatar. For example, “ the presence of the avatar robot contributed to a more enjoyable recess and promoted conversations with their peers.” Specifically, they commented, “It’s helpful to have the robot as a playmate during recess” and “After spending time with the robot, I found it easier to talk a lot with my classmates, and we became closer.” It has been suggested that comfortable recess time is crucial for building relationships and facilitating learning (Ramstetter et al., 2010). Therefore, the avatar robot is considered to contribute to providing children with a comfortable recess time that encourages interaction among them.

Finally, the consideration skill, indicating self-evaluation of one’s considerable attitude toward others, was improved in the classrooms where the avatar robot was installed. Considering that the avatar consistently exhibited considerate behavior toward children throughout the experimental period, this suggests the possibility that the avatar robot could serve as a positive role model for children. We found consistent answers in the free description that indicated children’s impressions, for example, “I want to learn from the robot and get better at saying ’thank you”’ and “I admire the robot’s reactions.” In addition, a child in the moral class who viewed the robot’s behavior positively had the following opinion: “The robot always responds to our presentations and interactions; we should adopt its behavior as well.” The results imply the necessity to carefully design the avatar’s characteristics to avoid unexpectedly influencing children’s behavior.

### 6.2 Teleoperator system

In the experiment, children in the classroom with the avatar robot reported a greater sense of comfort and perceived the robot as more helpful for their wellbeing. They also felt that the robot acted more independently. At the same time, there was no clear decreasing trend observed. Although the results do not suggest that having multiple operators harmed the children’s positive impression of the robot, we did not gain clear practical insights into which specific robot behaviors are most desirable for gaining children’s acceptance. Therefore, it would be valuable to conduct additional experiments with longer intervention periods in different classroom settings to see if similar results are observed. During this period, the proposed methods for remote operators to preshare dialogue content and information enabled avatar robots to maintain consistent speech and behavior. This facilitated seamless communication with children without causing severe discomfort. Thus, the children may not have noticed the switching of remote operators. Therefore, the practical feasibility of rotationally operating avatar robots by multiple individuals was demonstrated. Additionally, no comments were received from the children expressing any discomfort toward the avatar robot. By interacting with children remotely and providing consistent support and communication, avatar robots have demonstrated the potential to help students maintain positive relationships without causing stress, as intended to be checked in this experiment. Avatar robots, such as Sota (Nakanishi et al., 2022) and Telenoid (Yamazaki et al., 2012), shown promising outcomes in enhancing children’s communication and participation. Previous studies have been limited to specific classes or short-term experiments; however, our system enabled the robot to participate in whole classroom activities alongside students over an extended period, demonstrating the feasibility of providing continuous attention and support in the classroom. This study also highlights the potential for nonteacher operators to teleoperate with avatar robots while maintaining consistent behavior and avoiding negative reactions from students. The results suggest that this approach can effectively support children’s learning and school education.

In this study, we conducted an experiment in which an avatar robot was operated by multiple operators taking turns to provide all-day support. The results demonstrated that avatar robots can support children’s learning and social interaction even when operated in shifts by different individuals. This finding suggests that, in real-world implementations where it is difficult to ensure a single operator’s continuous involvement, adopting a multi-operator approach may be a viable solution. At the same time, while our study focused on confirming the feasibility of multi-operator use, it was not designed to directly compare avatar robots operated by a single individual with those operated by multiple people. Nevertheless, examining the potential advantages and disadvantages of multi-operator control is an important issue. We believe that confirming the feasibility of multi-operator support in this study lays the groundwork for future empirical investigations from this perspective. Furthermore, leveraging the resources of the local community to operate avatar robots provides a solution for addressing teacher shortages and expanding educational support systems.

### 6.3 Limitations and future works

In this study, children only in Class 5C had prior exposure to the robot before the experiment, which might promote children to be accustomed to it and influence their responses. However, the current study was not intended to separate the potential effect of the prior exposure from other effects. Furthermore, the length of the prior exposure to the avatar robot might influence how much it was accustomed to the children and positively evaluated. Therefore, the future work should adequately investigate this issue.

In the Discussion, we suggested the potential for the avatar robot to be perceived as a positive role model by children. However, the present study did not incorporate methods that allow for an in-depth analysis of children’s psychological responses related to role modeling. This limitation reflects the absence of psychological assessment tools in the study design. Future research should address this gap by exploring children’s internal responses in greater detail through interviews, behavioral observations, and the use of validated psychological scales.

Since this study was conducted with fifth-grade students in a Japanese elementary school, the generalizability of the findings to other age groups or cultural contexts remains limited. Children’s responses to avatar robots may vary depending on developmental stage and cultural background. To address this limitation, future research should expand the scope of investigation by conducting studies with students of different age groups and in diverse cultural or institutional settings. Such studies would help evaluate the adaptability of the system and identify necessary modifications for implementation in varied contexts—for example, simplified user interfaces, systems for receiving input from teachers, and alignment with local classroom practices and curricula. Furthermore, to address the shortage of teachers, it is necessary to reveal the technical challenges that may arise when people from various backgrounds and characteristics, such as expertise, genders, ages, traits, and handicaps/gifts, operate the system. However, the current results are limited to an experimental setting with a small number of operators—specifically, three females without expertise in education. This limitation makes it challenging to demonstrate that the proposed method functions in a manner that potential citizen confederates from a wide range of backgrounds can effectively assist teachers in providing rich education support to children.

Finally, we plan to enhance the avatar robot design by adding features that will allow for personalized interaction, such as recognizing individual students, remembering conversations and summarizing the history of the dialogue. The introduction of such features is expected to enhance support for children in diverse learning environments (Baxter et al., 2017). We believe integrating the avatar robot with other teaching tools will provide a clearer understanding of children’s learning progress, enabling more appropriate and responsive educational support. Furthermore, as Papakostas et al. (2021) has shown such personalization and integration improvements will contribute to a more flexible avatar design potentially beneficial for students with special needs whose requests are not adequately addressed by the current support system (Papakostas et al., 2021). In addition, we will consider different types of teleoperators, such as older adults interested in contributing to education and professional counselors with expertise in helping children with special needs, to explore the potential of the proposed methodology to promote attentive educational support throughout the community.

## 7 Conclusion

In this study, we reported a field experiment concerning questions for an application of an avatar robot on the school-community collaboration by introducing Sota100 to a classroom of an elementary school concerning two questions: “whether an avatar robot can be effectively operated by multiple remote operators to support children’s classroom activities” and “whether switching between multiple operators would negatively affect children’s perception of the avatar robot”. It was introduced with an information sharing method to maintain consistent communication with children despite changes in operators. Namely, this study expanded upon the setup of a previous study (Kawata et al., 2023) by introducing avatars operated by three operators in turns for 9 days, from 4 July 2022 to 14 July 2022, excluding holidays. The test was conducted in a fifth-grade class at a Mie University-affiliated elementary school. The field study conducted at elementary schools suggested that the continuous and daily use of an avatar robot, which actively engaged in the classroom by calling out names of children, encouraging their opinions, praising presenters, and freely interacting with children during recess time, could effectively provide comfortable classes and recess time for children. Additionally, it showed an improvement in their motivation to learn and communicate in the classroom with the avatar robot. Furthermore, the policy of listening to children without criticism may have led to a ripple effect, improving their consideration skills, possibly because the children perceived the avatar robot as a normative entity. Notably, the findings suggest that the children did not develop negative impressions of the avatar robot, even if an avatar robot with multiple remote operators is introduced to participate in whole classroom activities. To our knowledge, this is the first study to demonstrate the possibility that multiple people can take turns operating an avatar robot to participate alongside the children in the classroom activities without causing them any strong negative reactions, and that the avatar robot can support the children’s class activities. This may be because the multiple remote operators could maintain consistent statements and behaviors of the avatar by incorporating a method in which they obtained information about the children and shared the information along with the history of dialogues with each other in advance. The results suggest that multiple nonteacher operators can teleoperate with the avatar robot, which could support children’s school life by creating an atmosphere wherein children feel comfortable expressing their opinions in class and providing a comfortable recess time. However, certain limitations must be acknowledged regarding the scope of the present study. This study was conducted using one classroom and one avatar robot in a specific culture and educational context. While positive effects were observed in supporting children’s learning and classroom participation, it remains unclear whether similar results would be achieved in other environments or with different types of robots. Further field studies in different locations and countries are needed to validate the generalizability of the method.

In the future, we aim to explore the potential of our proposed method to facilitate community-wide, attentive educational support. This involves considering various types of remote operators, including older adults who may be interested in contributing to education and professional counselors with expertise to assist children with special needs. In addition, we plan to improve the avatar robot by adding features for personalized interaction and integration with teaching tools, aiming to better support diverse learners. These enhancements could benefit students with special needs by enabling more flexible avatar designs.

## Data Availability

The raw data supporting the conclusions of this article will be made available by the authors, without undue reservation.
